# Hand- and Object-Mouthing of Rural Bangladeshi Children 3–18 Months Old

**DOI:** 10.3390/ijerph13060563

**Published:** 2016-06-04

**Authors:** Laura H. Kwong, Ayse Ercumen, Amy J. Pickering, Leanne Unicomb, Jennifer Davis, Stephen P. Luby

**Affiliations:** 1 Department of Civil and Environmental Engineering, Stanford University, Stanford, CA 94305, USA; jennadavis@stanford.edu; 2 Division of Epidemiology, School of Public Health, University of California, Berkeley, CA 94720, USA; aercumen@berkeley.edu; 3 Woods Institute for the Environment, Stanford University, Stanford, CA 94305, USA; amyjanel@stanford.edu (A.J.P.); sluby@stanford.edu (S.P.L.); 4 International Centre for Diarrhoeal Disease Research, Dhaka 1212, Bangladesh; leanne@icddrb.org

**Keywords:** non-dietary ingestion, child behavior, mouthing, exposure factors, rural, Bangladesh

## Abstract

Children are exposed to environmental contaminants by placing contaminated hands or objects in their mouths. We quantified hand- and object-mouthing frequencies of Bangladeshi children and determined if they differ from those of U.S. children to evaluate the appropriateness of applying U.S. exposure models in other socio-cultural contexts. We conducted a five-hour structured observation of the mouthing behaviors of 148 rural Bangladeshi children aged 3–18 months. We modeled mouthing frequencies using 2-parameter Weibull distributions to compare the modeled medians with those of U.S. children. In Bangladesh the median frequency of hand-mouthing was 37.3 contacts/h for children 3–6 months old, 34.4 contacts/h for children 6–12 months old, and 29.7 contacts/h for children 12–18 months old. The median frequency of object-mouthing was 23.1 contacts/h for children 3–6 months old, 29.6 contacts/h for children 6–12 months old, and 15.2 contacts/h for children 12–18 months old. At all ages both hand- and object-mouthing frequencies were higher than those of U.S. children. Mouthing frequencies were not associated with child location (indoor/outdoor). Using hand- and object-mouthing exposure models from U.S. and other high-income countries might not accurately estimate children’s exposure to environmental contaminants via mouthing in low- and middle-income countries.

## 1. Introduction

Individuals can be exposed to chemical and biological contaminants through inhalation, dermal absorption, and ingestion. Mouthing, including touching hands and objects to the lips or placing them in the mouth, is an important pathway of exposure for young children. Mouthing exposure factors, such as mouthing frequency, total mouthing duration per hour, and duration of mouthing per contact, and other exposure factors related to the dermal, ingestion, and inhalation pathways of exposure, are published in the U.S. Environmental Protection Agency Exposure Factors Handbook [1] and the European Commission’s Joint Research Centre ExpoFacts’ website. Details on mouthing behaviors and the objects with which children interact, along with contaminant transfer efficiencies and concentrations, have been incorporated into deterministic and probabilistic models, such as LifeLine™, CARES^®^, Calendex™, ConsExpo, and SHEDS, to estimate exposure to contaminants, such as lead [2], arsenic [3,4,5], pesticides [6,7,8], and fecal matter [9] and to model the amount of soil children ingest [10].

Hand- and object-mouthing frequencies correlate with certain child-, household-, and community-level characteristics. In meta-analyses of hand- and object-mouthing studies on U.S. children, increasing child age and indoor location were positively correlated with both hand- and object-mouthing frequencies, but there was no association with sex [11,12]. Household-level factors may also influence the type and frequency of objects a child contacts and has the potential to mouth. For example, older houses may have more flaking paint, resulting in higher reported prevalence of mouthing paint chips [13]. Community-level characteristics, such as local climate and agricultural land use, may also influence the objects in the domestic environment that are available for mouthing. In the U.S., children in colder climates interacted with different objects than children in warmer climates [13,14], although urban *vs.* rural household location was not associated with mouthing frequency [15]. However, the more extreme difference between urban and rural settings in low-income countries may impact the mouthing frequency of particular items due to their availability. For example, children in rural areas of the U.S., may have more limited opportunities to interact with agricultural animals and soil than children in rural areas of low-income countries such as Bangladesh, where 94% of rural houses have domestic poultry or livestock [16], 72% have earthen floors [17], and 85% use plant material for fuel [18].

In addition to child-, household-, and community-level characteristics, cultural and caregiving practices, such as encouraging children to eat with hands or use a pacifier, may also influence children’s mouthing behaviors. Eating with hands instead of utensils is a normal behavior in many cultures. A child eating with her own hands or hand-fed by a caregiver would have higher rates of hand-mouthing and lower rates of utensil-mouthing. Hand-mouthing not related to eating may also become a habit in children who eat with their hands. In contrast, use of a pacifier may occupy a large portion of a child’s total mouthing time and preclude the mouthing of other objects. In the U.S., parents recorded that over an entire day children 0–18 months old mouthed pacifiers for an average of 108 min, substantially longer than the time they spent mouthing plastic toys (17 min), other objects (9 min) and teethers (6 min) [19].

The physical and socio-cultural context of households in high-income countries and low-income communities of low-income countries are markedly different. As such, comparing the mouthing behaviors of children in different countries may elucidate the impact of some of these contextual characteristics. Most exposure studies are in high-income countries [1,20,21]; we identified only three studies in low-income countries. One study of 23 Zimbabwean infants used semi-structued observation to record the total number of times that children touched or mouthed particular foods, toys, body parts, soil and feces during a six-hour observation period [22]. The authors also recorded whether the foods, toys, and body parts with which the child interacted were visibly dirty or not. In another study, 21 Peruvian children 0–60 months old were observed for 12 h to record their interaction with chicken feces in and around the house. Among all children there was a mean of 3.9 feces-to-mouth contacts and 2.9 feces-to-hand contacts during the entire observation period [18]. In rural Bangladesh, 216 children 7–30 months old were observed for geophagia [17]. During a five-hour observation, 38 (18%) children put soil, clay, mud, or sand in their mouths at least once. However, the study did not quantify the hourly frequency of mouthing, the level of detail necessary for use of exposure factors in exposure models such as SHEDS.

A difference in the frequency of children’s hand- and object-mouthing could result in different total exposure to environmental contaminants. The duration of mouthing may or may not be important for quantifying exposure, depending on the contaminant in question [23,24]. The clinical relevance of the differential dose depends on the importance of mouthing as a pathway of contaminant intake and the contaminant’s dose-response function. For situations in which the difference is clinically relevant, the accuracy of risk assessment would benefit from the use of both location-specific exposure factors and location-specific contaminant concentrations. Therefore, using exposure factors from populations very different from the study population may be inappropriate for modeling risk.

This study provides a detailed evaluation the hand- and object-mouthing frequencies of a relatively large number of children in a low-income country. The resulting non-dietary ingestion frequencies can be compared to values from high-income countries to assess the appropriateness of translating exposure factors values across different contexts. They can also be used to develop exposure models specifically for children in rural Bangladesh.

With this research, we seek to better understand mouthing activities of children in a low-income country in order to assess potential differences in non-dietary ingestion exposure due to different child-, household-, and community-level characteristics and socio-cultural practices. We asked three questions: (1) What is the frequency of mouthing hands, objects, food, water, soil, and feces among rural Bangladeshi children 3–18 months old? (2) What are the household and child characteristics associated with mouthing hands and other objects? (3) How much does the frequency of mouthing hands and other objects differ compared to children in high-income countries?

## 2. Methodology

### 2.1. Sampling Frame

Participating households were drawn from the 2012–2015 Wash Benefits study, a large cluster-randomized trial of water, sanitation, hygiene, and nutritional interventions in rural Bangladesh which enrolled pregnant women and followed their children after birth. The trial cluster-randomized households to receive a water, sanitation, hygiene, and/or nutrition intervention, singly or in combination, or to receive no intervention. In the sanitation intervention arm, households were provided a latrine, a sani-scoop for hygienic removal of feces, and a child potty. In the combined water, sanitation, and hygiene intervention arm, households received the sanitation intervention as well as a safe water storage container and chlorine tablets for water treatment, handwashing stations, and soapy water. Detailed description of the Wash Benefits trial is elsewhere [25].

For our hand- and object-mouthing study, we used simple random sampling to select 50 children from each of three arms of Wash Benefits: control (Wash Benefits *n* = 1440), sanitation (*n* = 720), and combined water, sanitation, and hygiene (*n* = 720). Field staff conducted structured observation of the child during one day in April or May 2014. If the selected child was absent from the household on the day of planned observation, he/she was replaced by the study child in the nearest neighboring household participating in the same study arm.

### 2.2. Microactivity Data Collection

**Figure 1:**
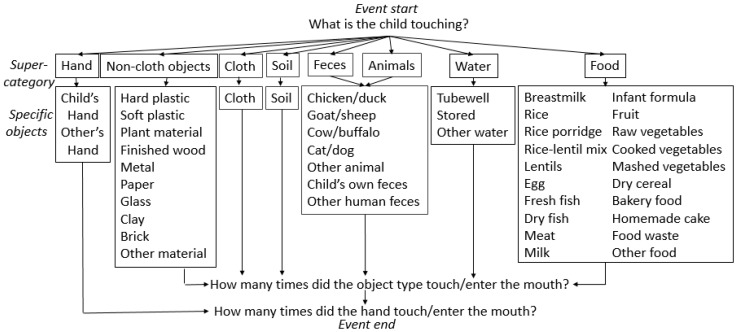
Schematic of structured observation method, including object super-category designation.

Following caregiver consent, trained staff observed a child’s activities during the daylight hours, varying the start time from 7 A.M. to noon and ending after five hours. The field staff recorded when the child began breastfeeding or sleeping but did not conduct observation during this time to respect privacy. Observations occurred on both weekdays and weekends. To conduct the structured observation, field staff recorded every object-to-mouth and hand-to-mouth contact as well as information on objects contacted by either hand. Observers prioritized recording mouth contacts over hand contacts. We defined mouthing to be contact of any individual’s hand or object with the child’s lips, inside of the mouth or tongue. We considered any contact of hands and non-food objects with the mouth as mouthing, and considered contact with food to be food intake. Observers detailed each hand or mouth contact with one or more of the following object super-categories [26]: hand/skin, soil, feces, animals, water, food, cloth and non-cloth objects. These object super-categories were sub-divided into specific objects (Figure 1). For example, “food” included *suji*, a rice flour or semolina porridge made with milk; *kitchuri*, a mixture of rice, lentils, and vegetables; fruit, and food waste (e.g., peelings). “Non-cloth objects” included plastic and metal objects, paper, wood furniture, glass, clay, bricks/concrete, and plant materials (e.g., sticks, leaves). “Soil” included both soil from outside and dust/soil from inside the home.

A new event was recorded when the child interacted with a new object(s). For example, if the child stopped playing with paper and began to touch soil and a hard plastic toy, the observer started a new event by answering the prompt “What is the child touching?” The observer recorded the number of times objects from the same super-category touched or entered the child’s mouth, but did not record the sequence in which objects contacted the mouth within a single event or the duration of mouthing events.

After the structured observation concluded, observers recorded child-specific information, such as the types of clothing and shoes worn during most of the observation, mobility and hand dominance (as observed), and time of day the child was inside, outside, breastfeeding and sleeping. In addition, they recorded household-specific information, such as the presence of feces in the courtyard area where the child spent time and the material of the household floor. All observational and survey data were recorded on a digital tablet using Open Data Kit software.

### 2.3. Observer Training

Five observers were trained over one week by practicing structured observation on video clips of children in the domestic environment. Structured observation records were carefully reviewed against the video clips to discuss errors before structured observation was conducted again on the same clips to improve accuracy. Observers also conducted two days of field piloting. For quality control during the study, some children were observed by multiple observers at the same time. For these observations, we assessed inter-observer reliability by calculating Krippendorf’s alpha on the number of mouthing contacts with each type of object. For field work to continue, we required inter-observer reliability to be high (Krippendorf’s alpha ≥ 0.80) [27]. We also evaluated the percent error between multiple observers by dividing the number of total mouthing contacts recorded by one observer by the average number of mouthing contacts recorded by all observers. After each observation, observers were questioned about records of contacts that were unusual in type or frequency. When there was more than one observation for the same child, one was randomly selected to include in the analysis of mouthing frequencies.

### 2.4. Statistical Analysis

Young children’s exposure is not limited to particular locations or times of day, so we calculated mouthing frequencies over the observed waking hours. We assume that the observed waking hours are representative of all of the child’s waking hours on the basis of one U.S. study that examined the behavior of 25 children throughout the day and found that a child’s rate of mouthing did not vary significantly by hour [28]. For each child, we summed all the contacts in the observation and divided by the total time the child was awake during the observation to yield contacts per waking hour. For location-specific contact frequencies, we divided the sum of mouthing contacts in each location (*i.e.*, inside *vs.* outside) by the number of hours observed in that location. Due to a technical error, full location data were collected for only 65 children (44%). Events for which no location was recorded were included in the location-independent analysis and excluded from the location-specific analysis.

According to EPA exposure assessment guidelines [29], we classified children into three age groups: 3 to <6 months (3–6 months old), 6 to <12 months (6–12 months old), and 12 to <24 months (12–24 months old); this study used the category 12–18 months because no child was older than 18 months. Although U.S. EPA age categories of child development have not been validated specifically in Bangladeshi children, using these age categories permits comparison with publicly available data. We examined household-level characteristics including years of mother’s formal education, number of people in the compound, number of children under five in the household and compound, and the compound-level ratio of people to children <5; child-level characteristics included child age, sex, and handedness. Data were right-skewed and analyzed with non-parametric statistical tests using the R packages *stat*, *irr* and *goftest*. We used Spearman’s rank correlation to assess the correlation between mouthing frequency and the aforementioned continuous variables. We used the Mann-Whitney U-test to assess whether or not the mouthing frequencies of groups that differed along a binary characteristic were from the same population. Similarly, we used the Kruskal-Wallis and Dunn tests to compare the mouthing frequencies of groups from that differed by demographic variables with more than two values.

In order to create continuous, smooth distributions for incorporation into probabilistic exposure models, we bootstrapped the hand- and object-mouthing frequency data. We then tested the null hypothesis that the data fit normal, lognormal, and Weibull distributions by using the Kolmogorov-Smirnov, Cramer-von-Mises, Anderson Darling, and chi-squared tests and visual inspection. We failed to reject the normal, lognormal, and Weibull distributions and ultimately decided to use age group-specific 2-paramter Weibull distributions to allow for comparison with meta-analyses of U.S. studies [11,12]. To determine the distributional parameters, we used maximum likelihood estimation with the initial parameters of scale = 20 and shape = 1 on 1000 iterations of data bootstrapped from the original dataset. We also used bootstrapped data to identify 95% confidence intervals. We then compared Weibull-distributed data from Bangladesh and the U.S. [11,12] with empirical data from Taiwan [20], and Australia [21].

### 2.5. Ethical Approval

Participants provided written, informed consent before the observation. The study was conducted in accordance with the Declaration of Helsinki and received ethics clearance from Stanford University, University of California-Berkeley, and the International Centre for Diarrhoeal Disease Research, Bangladesh (Protocol PR-11063).

## 3. Results

### 3.1. Inter-Observer Agreement

For the 17 children that were observed by two or three observers at the same time, Krippendorf’s alpha was 0.93. The total number of mouthing contacts for children who were observed by two or more observers differed by an average of 2.8% (standard deviation (SD) 4.9%).

### 3.2. Observational Results

Observers spent five hours at the homes of each of the 150 participating children. Data from two children were excluded from analysis because data were lost for one child and there was only one child younger than three months old in the study. The average age of the 148 children was 8.3 months; 56% could not crawl or walk. Housing quality was poor, with 54% of households lacking electricity, 62% having a tin roof, and 85% having floors of dirt. Ninety-nine percent of household courtyards were also made of dirt. There were an average of 12 people per compound, including an average of one child under <5 years old. Fecal matter was observed in all of the 148 household courtyards visited. Field staff observed poultry feces in 139 (94%), cow/buffalo feces in 37 (25%), goat/sheep feces in 13 (9%), other animal feces in 6 (4%) and human feces in 7 (5%) (Table 1).

**Figure 2:**
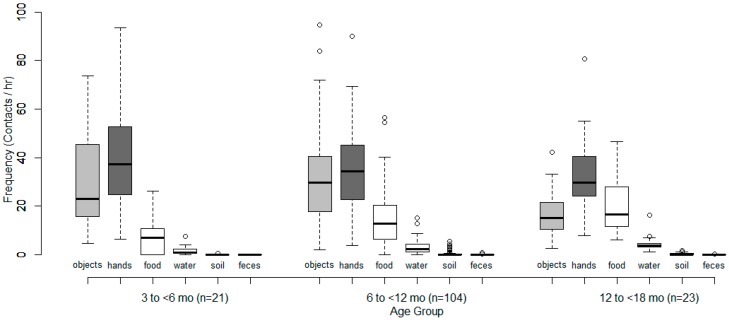
Observed hand- and object-mouthing frequencies by age group. Hands and objects are shaded for comparison across age groups. Objects include both cloth and non-cloth items.

Children were awake and not breastfeeding for an average of 3 h 56 min during the five-hour observation period. Children 3–6 months old mouthed hands a median of 37 times/h compared to 34 times/h for children 6–12 months and 30 times/h for children 12–18 months. Median hand-mouthing frequency decreased with increasing child age in months (r = 0.05, *p* < 0.01). Children 3–6 months old mouthed objects 23 times/h compared to 30 times/h for children 6–12 months and 15 times/h for children 12–18 months. There was not a monotonic trend by age group and a negligible decrease in median object-mouthing frequency with increasing child age in months (r = 0.08, *p* < 0.001) (Figure 2, with details in Appendix Table A1).

Approximately one-tenth (2/21) of children 3–6 months old, one-quarter (29/104) of children 6–12 months old, and one-third (8/23) of children 12–18 months old put soil in their mouths. These 39 children mouthed soil during 80 different events. In eight of these events, the child mouthed soil at least once but the exact number of time was not recorded; these missing values were excluded from frequency analysis (Appendix Table A2(A)).

During the observation period, 28 children (19%) were observed touching feces a total of 48 times. Ingestion of feces was rare: of the 71 girls and 77 boys observed, four girls (6%) and no boys (0%) put feces into their mouths. One nine-month-old girl directly consumed poultry feces twice and cow/buffalo feces once while another nine-month-old girl consumed poultry feces once. An eleven-month girl consumed cow/buffalo feces four times and a sixteen-month old girl consumed goat/sheep feces once (Appendix Table A2(B)).

The child’s location was recorded for 66% of the total observation time (587 h). Of the 65 children for whom full location data were recorded, on average 50% percent of their time was outdoors. Children 3–6 months old were outside 55% (SD 27%) of their observed waking hours, whereas children 6–12 months old were outside 50% (SD 27%) and children 12–18 months old, 39% (SD 30%). The location of the other 83 children was recorded a mean of 1.6 h of an average 4.0 h awake. Of this time, children spent 48% of their time outdoors.

Hand- and object-mouthing frequencies were not associated with child location, sex or handedness. They were also not associated with the household-level characteristics of mother’s years of formal education or number of children <5, or with the compound-level factors of total number of people, number of children <5 or ratio of people to number of children <5 (all *p* > 0.05). The households in this study were part of three different intervention arms, but frequency of hand-, object-, food-, water-, soil-, and feces-mouthing did not vary across the study arms (Appendix Table A3).

Combining the data from children of all ages and all study arms, the median frequency of indoor hand-mouthing was 31.7 contacts/h, which was not significantly different than the outdoor frequency of 29.1 contacts/h (*p* = 0.41). The median frequency of indoor object-mouthing was 23.1 contacts/h, which was not significantly different than the outdoor frequency of 22.3 contacts/h (*p* = 0.58). Nonetheless, to compare with published exposure factors, we segregated the data by location and age group.

The median hand-mouthing frequencies for Bangladeshi children both inside and outside were generally higher than for same-aged children in U.S. [11], Taiwan [20], and Australia [21]. Object-mouthing frequencies inside and outside were also higher than those for same-aged children in the U.S. [12], but lower than those recorded for children in Taiwan [20], and Australia [21] (Table 2).

**Figure 3:**
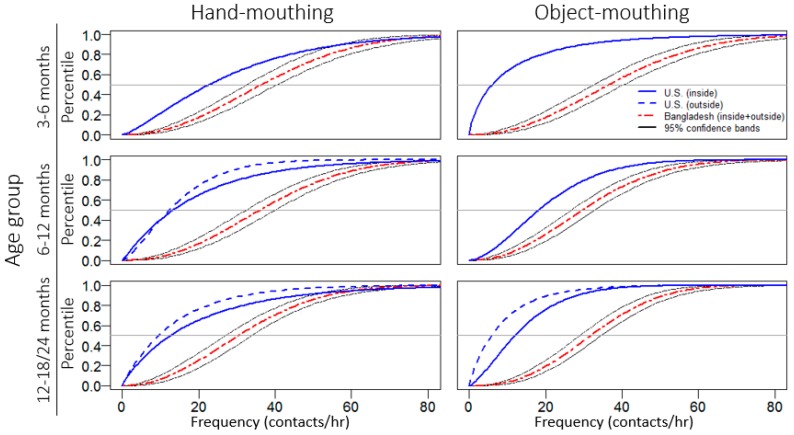
Cumulative distribution function for hand- and object-mouthing data by location and age group, for children from Bangladesh and the U.S. [11,12]. The Bangladeshi distribution is based on inside and outside frequencies combined. The U.S. distributions are separated by location; for some age groups the outside mouthing frequency distribution has not been determined. The oldest age group is 12–18 months for Bangladeshi children and 12–24 months for U.S. children.

**Figure A1:**
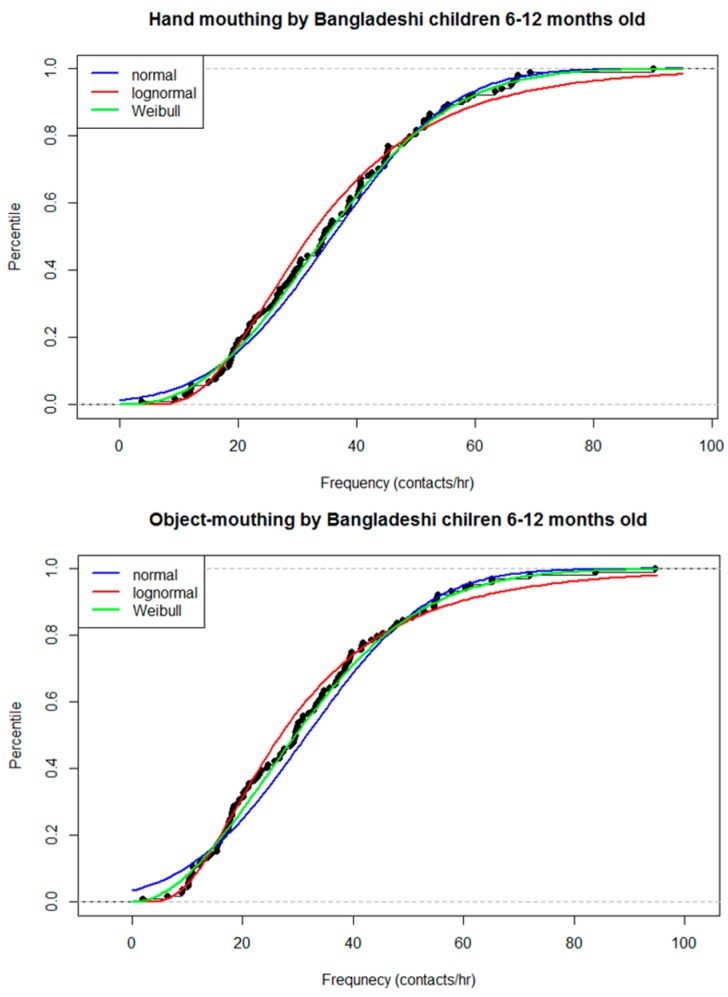
Comparison of normal, lognormal, and Weibull distributions against observed data.

We determined the appropriate distribution for modeling the mouthing frequency data by considering the 6–12 month age group, as this age group had considerably more observations than the other two. Testing for goodness-of-fit, we could not reject normal, lognormal or Weibull distributions (all *p* > 0.05); however, visually the Weibull distribution provided the best fit (Appendix Figure A1; Weibull shape and scale parameters are provided in Appendix Table A4). We graphically compared the Weibull distributions of mouthing frequencies for children in Bangladesh and the U.S., the only high-income country with available distributional data [11,12]. Since the difference between the indoor and outdoor mouthing frequencies of Bangladeshi children was not significant, we plot the distribution that represents mouthing in any location. The median frequencies at which young Bangladeshi children mouth hands and objects were between the 73rd to 95th percentiles of the mouthing frequency distributions for U.S. children (Figure 3). The median indoor hand-mouthing frequency of Bangladeshi children 6–12 months old was 2.3 times higher than their U.S. counterparts. The median outdoor object-mouthing frequency of Bangladeshi children 12–18 months old was 2.2 times higher than that of U.S. children 12–24 months old.

## 4. Discussion

Hand-mouthing accounted for over half of total mouthing. Median hand-mouthing frequencies of children in Bangladesh were 1.7 to 3.4 times higher than those published for children in the U.S. [11,12], Taiwan [20], and Australia [21]. Interpretation of these results should consider how each of the studies defined hand-mouthing, which can be divided into four categories: a child mouthing his own hand without involvement of food, a child mouthing his own hand while eating, a child mouthing another person’s hand without the involvement of food, and a child mouthing another person’s hand while being fed. The inclusion of eating and feeding periods during the observation may result in higher average hand- or object-mouthing frequencies than if eating and feeding periods are excluded because eating and feeding are accompanied with high frequencies of hand- and utensil-mouthing. Inclusion of mouthing another person’s hand may also increase total hand-mouthing.

In quantifying hand-mouthing, the largest study of the U.S. meta-analyses quantified children mouthing their own hands without the involvement with food; eating periods were excluded from observation with the rationale that during eating periods children cannot mouth non-food items [30]. This study neglects exposure that may be occurring during eating and feeding periods. While other U.S. studies included eating periods during observations and quantified children mouthing their own hands while eating [13,14,21,27,32,33,34], they excluded children’s interactions with other individuals, potentially underestimating total exposure.

In this study, we add depth to the understanding of children’s exposure through hand-mouthing by including all four categories of hand-mouthing—mouthing of children’s own hands and others’ hands, considering both dietary and non-dietary events. We have found only one other study, of children 7–12 years old, that is similarly inclusive [33]. Mouthing of children’s own hands while eating is an important element of total hand-mouthing [35], particularly for children who eat with their hands when young or in cultures where it is customary to eat by hand. Similarly, including mouthing of others’ hands during child feeding is important in contexts where caregivers feed children by hand, rather than with a utensil. In cultures where it is not common for children to eat or be fed by hand, the fraction of hand-mouthing while eating may be replaced with utensil-mouthing, which may also be relevant to a child’s exposure to particular contaminants. Further study into which of the four categories of hand-mouthing contributes the most to children’s exposure to contaminants could help inform the development of interventions. For example, if children’s mouthing of their own hands is identified as the most important transmission pathway for ingesting pathogens, a child handwashing intervention may be appropriate. In contrast, if children’s mouthing of caregiver hands is identified as the most important category due to the high frequency of caregivers putting their hands in children’s mouths during feeding, a suitable intervention might encourage caregivers to use utensils for feeding rather than their hands.

One hypothesis to explain the lower rate of hand- and object-mouthing among U.S. children is that U.S. children often use pacifiers whereas Bangladeshi children typically do not. Children who use pacifiers put them in their mouths infrequently, but each time mouth them for long periods of time, occupying their mouths and potentially reducing the frequency of mouthing non-pacifier objects. In one U.S. study, children 3–18 months old mouthed pacifiers 15%–63% of total non-dietary mouthing time [28]. In a study of Taiwanese children analyzing pacifier use [20], median hourly mouthing duration of children who used pacifiers was 16.22 min/h which was significantly longer than that of children who did not use pacifiers (2.80 min/h, *p* < 0.001) (unpublished data from [20]). On the other hand, there is evidence suggesting that pacifiers may not affect the mouthing frequency of non-pacifier objects. Mouthing duration and frequency of non-pacifier non-dietary objects was not significantly different between children who used pacifiers (4.70 min/h; 13.74 times/h) and those who did not (2.80 min/h; 9.60 times/h) (unpublished data from [20]). Studying the mouthing behaviors of a larger number of children who do and do not use pacifiers could clarify the effects of pacifiers on mouthing frequency and duration.

The frequency of mouthing objects was much higher than the frequency of mouthing/ingesting food, supporting the idea that non-dietary ingestion may be an important source of young children’s exposure to environmental contaminants [1]. In addition to mouthing objects, children also mouthed soil and feces, which may be particularly important vehicles of transmission for certain contaminants. The frequency of soil and feces mouthing may also be higher in Bangladesh than high-income countries where soil and feces are not as prevalent in the household environment. In the study area of rural Bangladesh, houses, floors, and spaces immediately outside the home were commonly made of dirt, and domestic animals often roam freely inside and outside, scattering feces that are not immediately cleared. Compared to high-income settings, children in this low-income setting would more frequently encounter, and potentially mouth, objects such as plant material, soil, and fecal matter.

The percentage of children 3–18 months old that directly ingested any soil (37/149, 25%) was somewhat higher than the 18% (38/216) observed in another study in rural Bangladesh [17] and greater than the 13% (3/21) observed in Zimbabwe [22] (Appendix Table A2(A)). Children were also observed touching soil and mouthing their hands before handwashing, indicating that indirect soil ingestion may substantially contribute to total soil ingestion. The frequency of soil ingestion was independent of location, emphasizing that both indoor dirt floors and outdoor bare soil may play an important role in a child’s exposure to soil contaminants. A study of 2755 households in Mexico found that children of households that received a cement floor intervention had a lower prevalence of parasites, diarrhea and anemia [36]. Covering dirt floors with concrete or plastic, which can be swept or wiped clean, may reduce children’s exposure to contaminants in soil and soil-related diseases.

Fecal matter, which may harbor enteric pathogens, is another contaminant present in the domestic environment of rural Bangladesh [37]. In addition to direct consumption of feces by children, the frequency of hand-to-mouth contact may be an important determinant in the total exposure to enteric pathogens. Using *E. coli* as an indicator of fecal contamination, one study in Tanzania compared a child’s ingested dose of fecal matter along two pathways and estimated that 97% of the total fecal matter ingested by a child was due to hand-mouthing, whereas only 3% was due to ingestion of water [9]. Considering the fecally-contaminated domestic environment of Bangladeshi children and their higher hand-mouthing frequencies, Bangladeshi children are likely exposed to higher levels of fecal contamination than children in high-income countries.

Children in this study spent approximately half of their waking hours outdoors, supplementing the data on outdoor mouthing behaviors that are currently limited by the brief amount of time the observed children from high-income countries spend outdoors. In one U.S. study, children spent a median of 88% of the observed time inside [32]. During the four-hour observational period of another U.S. study, 13 children never went outside and the remaining 30 spent 76% of their time inside [13]. In the Taiwanese study, children were inside a mean of 92.6% of the time [20]. In each of these studies, children differentially mouthed hands and objects depending on inside *vs.* outside location. In contrast, the frequency of mouthing hands and objects was not associated with location for the Bangladeshi children in this study. In rural Bangladesh, homes are usually single rooms, with outdoor food preparation and cooking areas, and outdoor water and latrine facilities. Caregivers often conduct domestic and agricultural tasks outside the home and bring young children with them. There is no strict division between indoor and outdoor environments, with earthen floors inside and bare ground outside, and domestic animals that roam freely through open doors [16]. Caregivers in low-income settings may also perceive environmental contamination to be less risky than caregivers in high-income settings and be less likely to stop children from mouthing potentially contaminated hands and objects while outdoors.

## 5. Limitations

There are several limitations to this study. Our study involved 148 children, a large sample relative to most other exposure studies quantifying mouthing behavior [1,20,21]. However, after age-stratification, the 3–6 month and 12–18 month age groups had fewer than 30 children each. These groups were unbalanced with regard to sex and may have been unbalanced on other unmeasured characteristics. Particularly in these two age groups, there was insufficient power to detect differences in mouthing frequency based on child- or household-level characteristics due to the small sample size. Larger studies of children in these age groups would be useful to confirm or refute these findings.

In this study we compared data collected using different observational methods over different time periods. Structured and video observations are forms of direct observation commonly used to collect hand- and object-mouthing data [1]. In structured observation, trained observers use a structured observation instrument to guide them in recording a child’s hand and/or mouth contacts in real time. In video observation, videographers record a child’s activities on video and trained analysts subsequently watch the video to record the child’s contacts, often employing a computer-aided video translation software such as the Virtual Timing Device™ [38]. To gather detailed micro-level activity time-series data regarding hand- and mouth-contacts, a video is usually translated two or three times [28,29,30], thus requiring three to four times longer to collect data than with structured observation. Due to the large number of children and the duration of observations we targeted in this study, we employed structured observation. We conducted the observation over five hours both to capture children’s morning and afternoon activity and to overcome reactivity to the observer noted in shorter observations [39]. U.S. studies used structured or video observation over 2–10 continuous hours or 5–60 min on different days at different times of day [11,12]. The Taiwanese study used a two-hour video observation, conducted in the morning for 62 of 66 children [20]. The Australian study used video observation during 10-min intervals at different times over multiple weeks [21]. Despite these differences, one study examining various methods suggests that comparing these diverse studies is appropriate. The method-comparison study examined the results of structured and video observation among 25 children and found that structured observation resulted in fewer recorded contacts and shorter mouthing duration than video observation, but these differences were not significant [28]. It also found that video observation produced less variance in the range of mouthing frequencies and durations observed [28]. Similarly, studies with shorter observation periods, such as the Australian study [21] are expected to have a higher variance in the observed mouthing frequencies.

This study was embedded in an intervention trial so the results may not be fully generalizable. While this analysis included children in the control arm of the intervention study, these children may not be representative of all rural Bangladeshi children as their participation in the control arm’s repeated household visits and health surveys may have increased the attentiveness of their caregivers or directly influenced their behavior, reducing the frequency of hand- and object-to-mouth contacts. Children in the control arm may display lower mouthing frequencies than non-study children, suggesting that our results may underestimate mouthing frequencies of the rural Bangladeshi population. Two-thirds of participating households were enrolled from intervention arms of an ongoing trial that aims to modify the domestic environment and the behavior of the child’s caregiver. The hardware and behavioral interventions may have altered the presence of objects in the domestic environment with which children could interact or altered the caregiver’s awareness of and attention to domestic and child hygiene. For example, the physical environment could be modified by reducing the number of child and animal feces through use of the sani-scoop. A caregiver may have also modified her behavior to encourage child handwashing or discourage a child from mouthing hands or objects. The difference in the median frequencies between study arms was small and not significant, varying from 0.0 contacts/h for soil- and feces-mouthing to 4.8 contacts/h for hand-mouthing, though the difference may not be significant due to the insufficient power of a relatively small sample size and short duration of observation.

While we did record exact counts for the number of times soil, water, hands, and cloth were mouthed, we did not record the number of times each particular object or food type was mouthed. Future studies that capture the number of contacts with specific object types will allow us to evaluate exposure to object- or food-specific toxins, such as diisononyl phthalate [40], a chemical in soft plastics that has caused adverse developmental and carcinogenic effects in rodents [41]. Future work could also compare the duration of mouthing between different child populations and evaluate the relative importance of frequency and duration in exposure to particular contaminants.

Due to a technical error, we did not record the locations of the child during the latter portion of the observation for some children who moved in and out frequently. If these children acted in a location-specific manner during the latter portion of the observation but not the former portion of the observation, then the location-specific analysis presented may be inaccurate. The time during the observation at which the location stopped being recorded was random and no one, including the observer, was aware of the error so it is unlikely that the child’s behavior changed due to the error.

## 6. Conclusions

Mouthing frequencies vary across age and geography and may also be related to household-level and cultural factors. Hand- and object-mouthing frequencies were different from those observed among children in high-income countries, and higher than those observed in the United States. Using exposure factor data from settings very different than the local settings of concern could over- or underestimate exposure to contaminants transmitted primarily through hand- and/or object-mouthing. In considering whether or not a study of locally-specific exposure factors should be conducted, it would be worthwhile for risk assessors to consider the relevant child-, household-, and community-level characteristics of the local situation and compare them with the characteristics of children studied previously. For example, risk assessors could consider children’s age and location, house floor material, and whether or not eating or feeding by hand is common. If the combination of characteristics is very different than those of children that have been previously studied, a location-specific study to determine appropriate exposure factors may be warranted.

Additionally, in this study of Bangladeshi children, hand-mouthing accounted for more than half of all mouthing. Further research to quantify the relative contribution of contamination transmitted through children’s mouthing of their own hands compared to their mouthing of other individuals’ hands could guide the design of interventions to reduce exposure.

## Figures and Tables

**Table 1:** Characteristics of sample participants, households, and compounds.

Characteristic	Mean (SD) or Frequency (%)			
	Total (*n* = 148)	3–6 Months (*n* = 21)	6–12 Months (*n* = 104)	12–24 Months (*n* = 23)
*Child characteristics*				
Male	77 (51.7)	13 (61.9)	53 (51.0)	11 (47.8)
Age in months	8.3 (3.1)	4.5 (0.7)	7.81 (1.5)	14.2 (1.4)
Mobility				
Sit only	82 (55.7)	21 (100.0)	60 (57.7)	1 (4.3)
Crawl	50 (33.6)	0 (0)	43 (41.3)	7 (30.4)
Walk	16 (10.7)	0 (0)	1 (1.0)	15 (65.2)
Hand dominance				
No dominant hand	80 (54.4)	9 (42.9)	55 (52.9)	16 (69.6)
Right hand dominant	61 (40.9)	12 (57.1)	44 (42.3)	5 (21.7)
Left hand dominant	7 (4.7)	0 (0)	5 (4.8)	2 (8.7)
*Household characteristics*				
Study arm (Number of households)				
Control	49 (32.9)	7 (33.3)	36 (34.6)	6 (26.1)
Sanitation	49 (33.6)	7 (33.3)	31 (29.8)	11 (47.8)
Water + sanitation + hygiene	50 (33.6)	7 (33.3)	37 (35.6)	6 (26.1)
Number of people in compound	12.2 (6.9)	13.4 (7.0)	12.8 (7.2)	8.8 (3.3)
Number of children <5 in compound	2.0 (1.1)	1.8 (1.2)	2.0 (1.2)	1.7 (0.8)
Number of children <5 in household	1.4 (0.6)	1.3 (0.7)	1.4 (0.6)	1.3 (0.6)
Mother’s years of formal education	5.8 (3.5)	5.0 (2.6)	5.6 (3.6)	7.7 (2.4)
Has electricity	84 (56.4)	13 (61.9)	57 (54.8)	14 (60.9)
Roof of tin	91 (61.7)	18 (85.7)	67 (64.4)	6 (26.1)
Owns a mobile phone	121 (81.9)	18 (85.7)	81 (77.9)	22 (95.7)
Household has dirt floor	126 (85.2)	20 (95.2)	91 (87.5)	15 (65.2)
Courtyard has dirt floor	146 (98.7)	21 (100.0)	103 (99.0)	22 (95.7)
Child feces observed in courtyard	7 (4.7)	0 (0)	5 (4.8)	2 (8.7)
Animal feces observed in courtyard	147 (99.3)	21 (100.0)	103 (99.0)	23 (100.0)

**Table 2:** Observed age- and location-specific (**A**) hand-mouthing and (**B**) object-mouthing frequencies from this study compared to observed and modeled results from previous studies in high-income countries.

**(A) Hand-Mouthing Frequency (Contacts/h)**											
**Demographics**			**Location**								
**Study**	**Country**	**Age Group**	**Inside**			**Outside**			**Inside + Outside**		
			***n***	**Mean**	**Median**	***n***	**Mean**	**Median**	***n***	**Mean**	**Median**
**This Study**	**Bangladesh**	**3–6 months**	**21**	**43.6**	**39.0**	**21**	**40.0**	**30.0**	**21**	**39.5**	**37.3**
Xue [11]	USA	3–6 months	23	28.0	23.0	-	-	-	-	-	-
Greene * [30]	USA	3–6 months	23	28.0	23.0	-	-	-	-	-	-
**This Study**	**Bangladesh**	**6–12 months**	**103**	**40.5**	**31.6**	**103**	**34.0**	**29.4**	**104**	**35.8**	**34.4**
Xue [11]	USA	6–12 months	119	18.9	14.0	10	14.5	11.6			
Greene * [30]	USA	6–12 months	88	19.8	14.5	-	-	-	-	-	-
Tulve^,^* [31]	USA	6–12 months	9	14.4	5.0	-	-	-	-	-	-
Black * [13]	USA	6–12 months	11	19.1	13.7	-	-	-	-	-	-
Beamer * [32]	USA	6–12 months	11	14.6	17.0	-	-	-	-	-	-
Tsou [20]	Taiwan	7–12 months	8	16.7	16.3	-	-	-	-	-	-
Brinkman [21]	Australia	5–12.5 months	-	-	-	-	-	-	22	13.1	9.9
**This Study**	**Bangladesh**	**12–18 months**	**23**	**38.7**	**28.2**	**23**	**30.9**	**23.1**	**23**	**32.3**	**29.7**
Xue [11]	USA	12–24 months	245	19.6	14.0	32	13.9	8.0	-	-	-
Greene * [30]	USA	12–24 months	123	20.1	14.0	-	-	-	-	-	-
Tulve^,^* [31]	USA	12–24 months	84	18.5	14.0	10	13.3	11.0	-	-	-
Black * [13]	USA	12–24 months	20	17.1	14.6	12	12.6	7.0	-	-	-
Tsou [20]	Taiwan	12–24 months	30	12.2	8.9	-	-	-	-	-	-
Brinkman [21]	Australia	12.5–20.5 months	-	-	-	-	-	-	20	23.3	8.2
**(B) Object-Mouthing Frequency (Contacts/h)**											
**This Study**	**Bangladesh**	**3–6 months**	**21**	**38.9**	**24.1**	**21**	**23.1**	**21.6**	**21**	**29.6**	**23.1**
Xue [12]	USA	3–6 months	19	11.2	9.3	-	-	-	-	-	-
Greene ^ [30]	USA	3–6 months	19	11.2	9.3	-	-	-	-	-	-
**This Study**	**Bangladesh**	**6–12** months	**103**	**33.7**	**26.6**	**103**	**30.0**	**25.6**	**104**	**31.6**	**29.6**
Xue [12]	USA	6–12 months	82	20.3	19.0	-	-	-	-	-	-
Greene ^ [30]	USA	6–12 months	82	20.3	19.0	-	-	-	-	-	-
Tulve ^ [31]	USA	6–12 months	9	72.3	84.0	-	-	-	-	-	-
Beamer ^ [32]	USA	6–12 months	11	43.9	38.6	-	-	-	-	-	-
Tsou [20]	Taiwan	7–12 months	8	65.7	60.7	-	-	-	-	-	-
Brinkman [21]	Australia	5 to 12.5 months	-	-	-	-	-	-	22	50.4	47.0
**This Study**	**Bangladesh**	**12–18 months**	**23**	**21.2**	**17.3**	**23**	**18.4**	**13.2**	**23**	**17.0**	**15.2**
Xue [12]	USA	12–24 months	137	14.2	12.3	21	8.8	6.0	-	-	-
Greene ^ [30]	USA	12–24 months	134	14.5	13.3	-	-	-	-	-	-
Tulve ^ [31]	USA	12–24 months	84	46.7	40.5	10	9.2	5.5	-	-	-
Beamer ^ [32]	USA	12–24 months	7	26.8	29.0				-	-	-
AuYeung ^ [26]	USA	12–24 months	-	-	-	7	7.5	7.9	-	-	-
Tsou [20]	Taiwan	12–24 months	30	18.5	11.8	-	-	-	-	-	-
Brinkman [21]	Australia	12.5–20.5 months	-	-	-	-	-	-	20	29.7	12.3

Objects include both cloth and non-cloth items. * = modeled with a Weibull distribution as reported in [11]; ^ = modeled with a Weibull distribution as reported in [12]; - means data have not been collected on children of the specified age and location.

## Appendix

**Table A1:** Frequency of mouthing hands, objects, foods, water, soil, and feces, by age group, for all locations combined.

Object Super-Category	Mouthing Frequency (Contacts/h)											
	Age Group											
	3–6 Months (*n* = 21)				6–12 Months (*n* = 104)				12–18 Months (*n* = 23)			
	Mean	Median	Min	Max	Mean	Median	Min	Max	Mean	Median	Min	Max
Hands	39.5	37.3	6.5	93.6	35.8	34.4	3.7	90.0	32.3	29.7	7.9	80.7
Objects	29.6	23.1	4.7	73.6	31.6	29.6	2.0	94.6	17.0	15.2	2.7	42.4
Cloth	10.9	10.4	3.4	24.7	9.3	8.3	0.0	45.1	5.1	3.9	1.7	13.4
Other objects	18.7	9.0	0.0	52.6	22.2	20.7	1.3	79.5	12.0	8.8	0.9	38.5
Dietary intake (mouthfuls)	6.8	6.9	0.0	26.2	14.5	12.8	0.0	56.6	20.0	16.6	6.1	46.5
Water ingestion (# of times)	1.6	0.7	0.0	7.4	3.2	2.4	0.0	15.1	4.6	3.8	1.1	16.3
Soil ingestion	0.1	0.0	0.0	0.7	0.3	0.0	0.0	5.5	0.3	0.0	0.0	1.6
Feces ingestion	0.0	0.0	0.0	0.0	0.0	0.0	0.0	1.0	0.0	0.0	0.0	0.2

**Table A2:** Comparison of age-specific (**A**) soil and (**B**) feces ingestion with other studies.

**(A) Soil Ingestion**							
**Study**	**Age Group**	**Mouthers**	**Children Observed**	**%**	**Child-Hours of Observation**	**Frequency (/h)**	
						**Mean**	**Median**
**This study**	**3–6 months**	**2**	**21**	**9.5**	**77**	**0.1**	**0**
Ngure [22]	3–6 months	1	7	14.3	42	1.7	-
**This study**	**6–12 months**	**29**	**104**	**27.9**	**420**	**0.3**	**0**
Ngure [22]	6–12 months	0	7	0	42	NA	NA
**This study**	**12–18 months**	**8**	**23**	**34.8**	**87**	**0.3**	**0**
Ngure [22]	12–18 months	2	7	28.6	42	2	-
**This study**	**3–18 months**	**39**	**148**	**26.4**	**590**	**1**	**0.5**
Ngure [22]	3–18 months	3	21	14.3	126	1.9	-
George [17]	6 to 30 months	38	216	17.6	1080	>0.2 times/h	
**(B) Feces Ingestion**											
**This study**	**3–6 months**	**0**	**21**	**0**	**77**	**0**	**0**
Ngure [22]	3–6 months	0	7	0	42	NA	NA
**This study**	**6–12 months**	**3**	**104**	**2.9**	**420**	**0**	**0**
Ngure [22]	6–12 months	1	7	14.3	42	0.2	-
**This study**	**12–18 months**	**1**	**23**	**4.3**	**90**	**0**	**0**
Ngure [22]	12–18 months	3	7	42.9	42	0.5	-
**This study**	**3–18 months**	**4**	**148**	**2.7**	**590**	**0.6**	**0.6**
Ngure [22]	3–18 months	4	21	19	126	0.3	-
George [17]	6 to 30 months	1	216	0.5	1080	> 0.2 times/h	
Marquis [18]	0–60 months	not given	21		120	0.3	-

%: number of children who ingested the object divided by the number of children observed.

**Table A3:** Mouthing frequencies, by study arm.

Object Super-Category	Median Frequency (Contacts/h)		
	Study Arm		
	Control	Sanitation	Water + Sanitation + Hygiene
	(*n* = 49)	(*n* = 49)	(*n* = 50)
Hands	35.8	34.6	31.0
Objects	21.9	25.2	27.1
Food	12.3	13.8	10.3
Water	2.4	2.8	2.3
Soil	0.0	0.0	0.0
Feces	0.0	0.0	0.0

**Table A4:** Weibull distribution parameters for hand- and object-mouthing frequencies.

Item Mouthed/Location		Age Group					
		3–6 Month		6–12 Month		12–18 Month	
		Shape	Scale	Shape	Scale	Shape	Scale
Hand	Inside	0.79	41.08	0.57	32.92	0.31	21.05
	Outside	1.29	43.73	0.66	30.52	0.83	29.22
Object	Inside	0.37	24.12	0.6	27.62	0.41	14.44
	Outside	1.67	26.06	0.65	26.5	0.76	16.62

## References

[1] U.S. Environmental Protection Agency (2011). Exposure Factors Handbook: 2011 Edition (EPA/600/R-090/052F).

[2] Canales R.A., Leckie J.O. (2007). Application of a stochastic model to estimate children’s short-term residential exposure to lead. Stoch. Environ. Res. Risk Assess..

[3] Hemond H.F., Solo-Gabriele H.M. (2004). Children’s Exposure to Arsenic from CCA-Treated Wooden Decks and Playground Structures. Risk Anal..

[4] Zartarian V.G., Xue J., Özkaynak H., Dang W., Glen G., Smith L., Stallings C. (2006). A Probabilistic Arsenic Exposure Assessment for Children Who Contact CCA-Treated Playsets and Decks, Part 1: Model Methodology, Variability Results, Model Evaluation. Risk Anal..

[5] Xue J., Zartarian V.G., Özkaynak H., Dang W., Glen G., Smith L., Stallings C. (2006). A probabilistic arsenic exposure assessment for children who contact chromated copper arsenate (CCA)-treated playsets and decks, Part 2: Sensitivity and uncertainty analyses. Risk Anal..

[6] Beamer P.I., Canales R.A., Ferguson A.C., Leckie J.O., Bradman A. (2012). Relative Pesticide and Exposure Route Contribution to Aggregate and Cumulative Dose in Young Farmworker Children. Int. J. Environ. Res. Public Health.

[7] Zartarian V., Xue J., Glen G., Smith L., Tulve N., Tornero-Velez R. (2012). Quantifying children’s aggregate (dietary and residential) exposure and dose to permethrin: Application and evaluation of EPA’s probabilistic SHEDS-Multimedia model. J. Expo. Sci. Environ. Epidemiol..

[8] Young B.M., Tulve N.S., Egeghy P.P., Driver J.H., Zartarian V.G., Johnston J.E., Delmaar C.J.E., Evans J.J., Smith L.A., Glen G. (2012). Comparison of four probabilistic models (CARES, Calendex, ConsExpo, and SHEDS) to estimate aggregate residential exposures to pesticides. J. Expo. Anal. Environ. Epidemiol..

[9] Mattioli M.C. M., Davis J., Boehm A.B. (2015). Hand-to-Mouth Contacts Result in Greater Ingestion of Feces than Dietary Water Consumption in Tanzania: A Quantitative Fecal Exposure Assessment Model. Environ. Sci. Technol..

[10] Özkaynak H., Xue J., Zartarian V.G., Glen G., Smith L. (2011). Modeled Estimates of Soil and Dust Ingestion Rates for Children. Risk Anal..

[11] Xue J., Zartarian V., Moya J., Freeman N., Beamer P., Black K., Tulve N., Shalat S. (2007). A Meta-Analysis of Children’s Hand-to-Mouth Frequency Data for Estimating Nondietary Ingestion Exposure. Risk Anal..

[12] Xue J., Zartarian V., Tulve N., Moya J., Freeman N., Auyeung W., Beamer P. (2010). A meta-analysis of children’s object-to-mouth frequency data for estimating non-dietary ingestion exposure. J. Expo. Sci. Environ. Epidemiol..

[13] Black K., Shalat S.L., Freeman N.C., Jimenez M., Donnelly K.C., Calvin J.A. (2005). Children’s mouthing and food-handling behavior in an agricultural community on the US/Mexico border. J. Expo. Anal. Environ. Epidemiol..

[14] Freeman N.C., Jimenez M., Reed K., Gurunathan S., Edwards R., Roy A., Adgate J.L., Pellizzari E.D., Quackenboss J., Sexton K. (2001). Quantitative analysis of children’s microactivity patterns: The Minnesota Children’s Pesticide Exposure Study. J. Expo. Anal. Environ. Epidemiol..

[15] Freeman N.C., Hore P., Black K., Jimenez M., Sheldon L., Tulve N., Lioy P.J. (2005). Contributions of children’s activities to pesticide hand loadings following residential pesticide application. J. Expo. Anal. Environ. Epidemiol..

[16] Parvez S.M., Kwong L.H., Rahman M., Ercumen A., Pickering A., Luby S., Ghosh P.K., Unicomb L. (2015). *E. coli* contamination of complementary foods and associations with domestic hygiene in rural Bangladesh.

[17] George C., Oldja L., Biswas S., Perin J., Lee G., Kosek M., Sack S., Ahmed S., Haque R., Parvin T. (2015). Geophagy is Associated with Environmental Enteropathy and Stunting in Children in Rural Bangladesh. Am. J. Trop. Med. Hyg..

[18] Marquis G.S., Ventura G., Gilman R.H., Porras E., Miranda E., Carbajal L., Pentafiel M. (1990). Fecal contamination of shanty town toddlers in households with non-corralled poultry, Lima, Peru. Am. J. Public Health.

[19] Juberg D.R., Alfano K., Coughlin R.J., Thompson K.M. (2001). An observational study of object mouthing behavior by young children. Pediatrics.

[20] Tsou M.-C., Özkaynak H., Beamer P., Dang W., Hsi H.-C., Jiang C.-B., Chien L.-C. (2015). Mouthing activity data for children aged 7 to 35 months in Taiwan. J. Expo. Sci. Environ. Epidemiol..

[21] Brinkman S., Gialamas A., Jones L., Edwards P., Maynard E. (1999). Child Activity Patterns for Environmental Exposure Assessment in the Home.

[22] Ngure F.M., Humphrey J.H., Mbuya M.N.N., Majo F., Mutasa K., Govha M., Mazarura E., Chasekwa B., Prendergast A.J., Curtis V. (2013). Formative Research on Hygiene Behaviors and Geophagy among Infants and Young Children and Implications of Exposure to Fecal Bacteria. Am. J. Trop. Med. Hyg..

[23] Cohen Hubal E.A., Sheldon L.S., Burke J.M., McCurdy T.R., Berry M.R., Rigas M.L., Zartarian V.G., Freeman N.C. (2000). Children’s Exposure Assessment: A review of factors influencing children’s exposure, and the data available to characterize and assess that exposure. Environ. Health Perspect..

[24] Cohen Hubal E.A., Suggs J.C., Nishioka M.G., Ivancic W.A. (2005). Characterizing residue transfer efficiencies using a fluorescent imaging technique. J. Expo. Anal. Environ. Epidemiol..

[25] Arnold B.F., Null C., Luby S.P., Unicomb L., Stewart C.P., Dewey K.G., Ahmed T., Ashraf S., Christensen G., Clasen T. (2013). Cluster-randomised controlled trials of individual and combined water, sanitation, hygiene and nutritional interventions in rural Bangladesh and Kenya: The WASH Benefits study design and rationale. BMJ Open.

[26] AuYeung W., Canales R.A., Beamer P., Ferguson A.C., Leckie J.O. (2004). Young Children’s Mouthing Behavior: An Observational Study via Videotaping in a Primarily Outdoor Residential Setting. J. Child. Health.

[27] Krippendorff K. (2004). Content Analysis: An Introduction to Its Methodology.

[28] Smith S.A., Norris B. (2003). Reducing the risk of choking hazards: Mouthing behavior of children aged 1 month to 5 years. Inj. Control Saf. Promot..

[29] U.S. Environmental Protection Agency (2005). Guidance on Selecting Age Groups for Monitoring and Assessing Childhood Exposures to Environmental Contaminants.

[30] Greene M.A. (2002). Mouthing Times amoung Young Children fron Otservational Data.

[31] Tulve N.S., Suggs J.C., McCurdy T., Cohen Hubal E.A., Moya J. (2002). Frequency of mouthing behavior in young children. J. Exp. Anal. Envrion. Epidemiol..

[32] Beamer P., Key M.E., Ferguson A.C., Canales R.A., Auyeung W., Leckie J.O. (2008). Quantified activity pattern data from 6 to 27-month-old farmworker children for use in exposure assessment. Environ. Res..

[33] Beamer P.I., Luik C.E., Canales R.A., Leckie J.O. (2012). Quantified outdoor micro-activity data for children aged 7–12-years old. J. Expo. Sci. Environ. Epidemiol..

[34] Reed K.J., Jimenez M., Freeman N.C., Lioy P.J. (1999). Quantification of children’s hand and mouthing activities through a videotaping methodology. J. Expo. Anal. Environ. Epidemiol..

[35] Akland G.G., Pellizzari E.D., Hu Y., Roberds M., Rohrer C.A., Leckie J.O., Berry M.R. (2000). Factors influencing total dietary exposures of young children. J. Expo. Anal. Environ. Epidemiol..

[36] Cattaneo M.D., Galiani S., Gertler P.J., Martinez S., Titiunik R. (2009). Housing, Health, and Happiness. Am. Econ. Assoc..

[37] Julian T.R., Islam M.A., Pickering A.J., Roy S., Fuhrmeister E.R., Ercumen A., Harris A., Bishai J., Schwab K.J. (2015). Genotypic and Phenotypic Characterization of *Escherichia coli* Isolates from Feces, Hands, and Soils in Rural Bangladesh via the Colilert Quanti-Tray System (IDEXX). Appl. Environ. Microbiol..

[38] Zartarian V.G., Ferguson A.C., Leckie J.O. (1997). Quantified dermal activity data from a four-child field study. J. Expo. Anal. Environ. Epidemiol..

[39] Ruel M.T., Arimond M. (2002). Spot-check Observational Method for Assessing Hygiene Practices: Review of Experience and Implications for Programmes. J. Health Popul. Nutr..

[40] Greene M.A. (2002). Mouthing Times and DINP Risk for Children over Three Years of Age.

[41] U.S. Consumer Product Safety Commission (2001). Chronic Hazard Advisory Panel on Diisononyl Phthalate (DINP): Report to the U.S. Consumer Product Safety Commission.

